# The Relationship between Impulsivity and Internet Gaming Disorder in Young Adults: Mediating Effects of Interpersonal Relationships and Depression

**DOI:** 10.3390/ijerph15030458

**Published:** 2018-03-06

**Authors:** Hyera Ryu, Ji-Yoon Lee, Aruem Choi, Sunyoung Park, Dai-Jin Kim, Jung-Seok Choi

**Affiliations:** 1 Department of Psychiatry, SMG-SMU Boramae Medical Center, Seoul 07061, Korea; hyera.ryu12@gmail.com (H.R.); idiyuni91@gmail.com (J.-Y.L.); choiar90@gmail.com (A.C.); spark.37eme@gmail.com (S.P.); 2 Department of Psychiatry, Seoul St. Mary’s Hospital, College of Medicine, The Catholic University of Korea, Seoul 06591, Korea; kdj922@catholic.ac.kr; 3 Department of Psychiatry and Behavioral Science, Seoul National University College of Medicine, Seoul 03080, Korea

**Keywords:** internet gaming disorder, impulsivity, depression, interpersonal relationships, serial mediation

## Abstract

*Background:* This study aimed to explore relationships between impulsivity, interpersonal relationships, depression, and Internet Gaming Disorder (IGD) symptoms. *Methods:* A total of 118 young adults participated in this study: 67 IGD patients who met five or more of the DSM-5 diagnostic criteria for IGD and 56 healthy controls. We administered questionnaires to assess IGD symptoms (Young’s Internet Addiction Test; Y-IAT), impulsivity (Barratt Impulsiveness Scale; BIS-11), interpersonal relationship (Relationship Change Scale; RCS), and depression (Beck Depression Inventory; BDI). We used PROCESS macro in SPSS to perform mediation analysis. *Results:* IGD symptom was positively related to depression and impulsivity, and negatively related to the quality of interpersonal relationships. Mediation analysis revealed full mediation effects of interpersonal relationships and depression on the association between impulsivity and IGD symptoms in the IGD group. Specifically, even after adjusting for gender as a covariate, high impulsivity was associated with greater difficulty with interpersonal relationships; which further affected depression and increased the risk of IGD. *Conclusions:* These results demonstrate the importance of early intervention in IGD patients, particularly in young adults with high impulsivity. When intervening in adults’ IGD, we should consider not only individual factors (e.g., depression) but also socioenvironmental factors (e.g., interpersonal relationships).

## 1. Introduction

Internet Gaming Disorder (IGD) is a kind of behavioral addiction that has been defined as a loss of control, and persistent and recurrent use of internet games leading to significant impairment in psychosocial functioning [1]. In particular, Diagnostic and Statistical Manual of Mental Disorders-Fifth Edition (DSM-5) has added an IGD as one of the conditions for further study in Section 3 [1], due to increased social interest worldwide. In previous studies, the prevalence of IGD, diagnosed according to DSM-5 criteria, was 1.16% in Germany [2], 2.5% in Slovenia [3], and 2.9% in Hungary [4]. However, higher estimates of prevalence (5.9% [5] and 10.8% [6]), have been reported in Korea. Because the risk of IGD is high in Korea, there is an urgent need to explore the characteristics of IGD in Korea, with further research in particular with respect to personal and environmental factors. 

Choi [7] and Choi et al. [8], who proposed an integrated pathway model of IGD based on the model of gambling problems developed by Blaszczynski and Nower [9], suggested that IGD is a complex disorder caused by interactions among different bio-psycho-social factors. One of the biological factors is trait impulsiveness. The impulsivity is a tendency to behave voluntarily with little or no prior consideration of consequences [10], and could be measured by the Barratt Impulsiveness Scale (BIS-11), which is one of the oldest and most widely used a measurement of impulsive personality traits. In the BIS-11, impulsivity could be classified cognitive, motor, and nonplanning impulsiveness. Cognitive impulsiveness is the propensity to respond or make decisions during problem solving without thinking; motor impulsiveness is the tendency of impulsive behaviors, such as manifesting difficulty with impulse control or acting without thinking. Finally, nonplanning impulsiveness is a lack of foresight and a tendency not to plan or consider consequences before starting something [11]. In many previous studies, impulsivity has been regarded as a marker for vulnerability to IGD [12,13,14,15,16]. Dalbudak et al. found that IGD was correlated with the severity of impulsivity among Turkish university students, and Lee et al. showed that trait impulsivity is a vulnerable factor of IGD in young adults. In particular, impulsivity assessed by neuropsychological tests, such as the Stop Signal Test and Go/No-Go Task, was related to IGD symptoms [13,17,18]. In addition, several studies showed that impulsivity is related to depression [19,20] and that it indirectly predicts loneliness and poor interpersonal relationships [21]. Thus, impulsivity is not only a core feature of IGD symptoms but also an important factor affecting individuals’ emotional and social functioning.

The quality of interpersonal relationships such as deficient social support and loneliness is among the social factors reported to be risk factors for IGD [22,23,24]. Previous studies have shown that loneliness increases the difficulty of maintaining healthy social interactions and may increase a preference for online social interaction, which can lead to IGD [22,25,26,27,28]. Furthermore, social contacts with family and friends help to reduce IGD symptoms [24,29]. In previous studies, loneliness, lack of social support, and lack of a sense of belonging have been shown to predict depression [30,31]. Thus, IGD could be assumed to be related to poor interpersonal relationships, which is a risk factor for depression and IGD symptoms.

Depression is one of the psychological factors associated with IGD. Prior research has shown that depression is a psychiatric disorder that is often comorbid with IGD [29,32,33]. In one study, 7% of adult IGD patients had a comorbid dysthymic disorder [34], and Ko et al. [35] identified a relationship between IGD and major depressive disorder or dysthymic disorder in college students. However, some studies have reported inconsistent results regarding the relationship between depression and IGD symptoms [29,36,37,38,39,40]. Ha et al. [41] suggested that participants who reported depression tended to seek cyberspace to avoid negative emotions and difficulties in daily life, and they had a high likelihood of being addicted to internet games because of the emotional support they found in cyberspace. Furthermore, a 2-year longitudinal study found that participants who overused internet games tended to be more depressed than those who did not [42]. Given these inconsistent results, it is necessary to clarify the relationship between depression and IGD. In addition, no previous studies have examined the relationship between bio-psycho-social factors and IGD symptoms by distinguishing between IGD and HC groups. Thus, it is important to examine the pathways connecting impulsivity, interpersonal relationships, depression, and IGD symptoms.

This study aimed to clarify relationships between impulsivity, interpersonal relationships, depression, and IGD symptoms and to identify the mediating effects of interpersonal relationships and depression on the relationship between impulsivity and IGD symptoms by distinguishing between IGD and HC group. We hypothesized that participants in the IGD group who also showed higher impulsivity would have more difficulty in interpersonal relationships, which would increase depression and increase the risk of IGD symptoms.

## 2. Materials and Methods

### 2.1. Participants and Procedure

A total of 123 young adults participated in this study, including 67 patients diagnosed with IGD and 56 healthy controls. The patients with IGD were seeking treatment at the outpatient clinics of SMG-SNU Boramae Medical Center in Seoul due to excessive internet gaming. They were diagnosed with IGD by a clinically experienced psychiatrist according to the DSM-5 criteria (more than five items). Also, the Structured Clinical interview was administered by a psychiatrist to identify past and current psychiatric disorders and only individuals with a history of intellectual disability or psychotic disorder were excluded. Of 67 patients with IGD, 10 had major depressive disorders, and two and one displayed social anxiety disorder and bipolar I disorder, respectively. Healthy controls (HC), who were recruited through advertisements, had no history of any psychiatric disorder. 

To screen for the participants’ intelligence quotient (IQ), the Korean-Wechsler Adult Intelligence Scale-IV (K-WAIS-IV) was administered, and five subjects with an IQ < 80 were excluded. Thus, 118 participants were included in the final analyses, including 62 in the IGD (male = 55; age = 25.54 ± 5.29 years) and 56 in the HC group (male = 38; age = 24.23 ± 3.92 years). All subjects completed informed consent forms before participating in the study. This study was conducted in accordance with the Declaration of Helsinki, and the protocol was approved by the Institutional Review Board of the SMG-SNU Boramae Medical Center (16-2014-139).

### 2.2. Measures

#### 2.2.1. Demographic Variables

All participants answered a questionnaire to provide basic information such as age, gender, education year, and Internet use time (weekday and weekend).

#### 2.2.2. Young’s Internet Addiction Test (Y-IAT)

The Y-IAT was developed by Young (1998) [28], and has been validated in Korea [43]. It is 20-item self-report questionnaire and each item was answered using a 5-point scale ranging from 1 (very rarely) to 5 (very frequently). The total score ranged from 20 to 100, with higher scores reflecting a greater tendency of IGD symptoms. Cronbach’s alpha was 0.96 in this study.

#### 2.2.3. Barratt Impulsiveness Scale-11 (BIS-11)

The BIS-11 is an 11-item, revised version of the original Barratt Impulsiveness Scale, which is used to assess the degree of impulsivity [11]. This scale includes three subscales: cognitive, motor, and nonplanning impulsiveness. Cronbach’s alpha was 0.76 in this study.

#### 2.2.4. Relationship Change Scale (RCS)

The RCS consists of 25-item and 5-point Likert scale, which was originally developed by Schlein et al. [44], and was later translated into Korean by Mun (1980) and revised according to the Korean culture by Chun [45]. The RCS measures interpersonal relationships, and higher scores indicate better interpersonal relationships. The total score ranges from 25 to 125. Cronbach’s alpha was 0.93 in this study.

#### 2.2.5. Beck Depression Inventory-II (BDI-II)

The BDI, developed by Beck et al. [46], is a 21-item self-report questionnaire that measures the severity of particular symptoms experienced over the past week. Total scores range from 0 to 63, and higher scores reflect more severe depression. The BDI-II has previously been validated in Korean [47]. Cronbach’s alpha was 0.92 in this study.

### 2.3. Statistical Analysis

Chi-square and *t*-tests were performed to compare the demographic and clinical characteristics of the HC and IGD groups. Pearson’s correlation analysis was conducted to examine relationships between IGD symptoms (Y-IAT), impulsivity (BIS-11), depression (BDI), and interpersonal relationships (RCS) in the HC and IGD groups, respectively. To examine whether the quality of interpersonal relationships and depression mediated the relationship between impulsivity and IGD symptoms, we performed serial mediation analysis using the SPSS PROCESS macro, version 2.16 (model 6), developed by Hayes [48]. Serial mediation assumes a causal chain linking the mediators, with a specified direction of causal flow [49]. In particular, we analyzed using bootstrapping method, because there were limitations of Sobel’s test (e.g., need a large sample). 5000 bootstrapping was used to identify indirect effects in the mediation models and analyzed with 95% confidence interval. SPSS software version 21.0 (SPSS, Inc., Chicago, IL, USA) was used for all data analyses.

## 3. Results

### 3.1. Demographic and Clinical Characteristics

In the total group, the mean age was 24.92 ± 4.71 years, and 78.8% (*n* = 93) of the sample were male. A comparison of the demographic and clinical characteristics of the IGD and HC groups showed that the percentage of males [*x*^2^(1) = 7.662, *p* < 0.05], the internet gaming use time on weekdays [*t*(69.23) = 9.088, *p* < 0.001], internet gaming use time on weekends [*t*(71.52) = 10.979, *p* < 0.001], Y-IAT scores [*t*(99.305) = 9.855, *p* < 0.001], BIS-11 scores [*t*(116) = 4.673, *p* < 0.001], and BDI scores [*t*(89.97) = 6.261, *p* < 0.001] were significantly higher in the IGD than in the HC group, and the RCS score [*t*(108.75) = −5.033, *p* < 0.001] was significantly lower in the IGD group. Results are shown in Table 1.

### 3.2. Association between IGD Symptoms and Clinical Variables in the IGD and HC Groups

In both IGD and HC group, IGD symptoms as measured by Y-IAT were significantly correlated with depression (IGD: *r* = 0.472, *p* < 0.001; HC: *r* = 0.363, *p* < 0.001), and interpersonal problems (IGD: *r* = −0.285, *p* < 0.05; HC: *r* = −0.268, *p* < 0.05). However, there was a significant relationship between IGD symptoms and impulsivity only in the IGD group (*r* = 0.306, *p* < 0.05), specifically, IGD symptoms were related to cognitive impulsiveness (*r* = 0.375, *p* < 0.001) and nonplanning impulsiveness (*r* = 0.275, *p* < 0.05), but not to motor impulsiveness (*r* = 0.129, *p* = 0.318). In the HC group, impulsivity was not related to the IGD symptoms (Table 2).

### 3.3. Relationships between Impulsivity, Interpersonal Relationships, Depression, and IGD Symptoms

**Figure 1:**
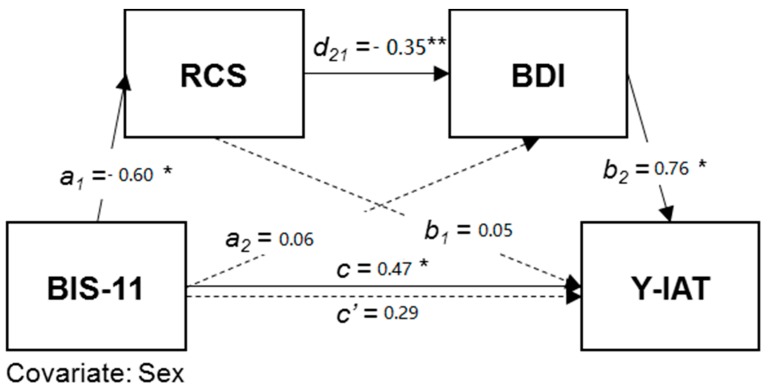
Serial mediation effects on problematic internet use in IGD group (*n* = 61). BIS-11 = Barratt Impulsiveness Scale; RCS = Relationship Change scale; BDI = Beck Depression Inventory; Y-IAT = Young’s Internet Addiction Test; Solid lines showed significant paths with standardized path coefficients and dashed line represents a nonsignificant path. *c*’ means direct effect of impulsivity (X) on internet addiction (Y) and *c* means indirect effect of impulsivity (X) on internet addiction (Y) through interpersonal relationship (M1) and depression (M2) in serial. Results showed that high impulsivity affected more difficulty in interpersonal relationships, which further affected depression and increased the risk of internet gaming disorder symptoms in IGD group. * *p* < 0.05, ** *p* < 0.001.

The model depicting serial mediation of the relationship between impulsivity and IGD symptoms by interpersonal relationships and depression in the IGD group is shown in Figure 1. We added sex as a covariate in the model. The serial mediation model was significant [*F*(2,58) = 5.9481, *p* < 0.05] and explained about 17% of the variance in IGD symptoms in the IGD group. Specifically, both the total effect of impulsivity on IGD (*c* = 0.47, SE = 0.19, *t* = 2.39, *p* < 0.05) and the direct effect of impulsivity on interpersonal relationships (*a*_1_ = −0.60, SE = 0.17, *t* = −3.39, *p* < 0.05) as mediating variables were significant. However, there was no significant direct effect of impulsivity on depression (path *a*_2_ in Figure 1). The direct effect of interpersonal relationships, the first mediating variable, on depression (*d*_21_ = −0.35, SE = 0.06, *t* = −5.53, *p* < 0.001), the second mediating variable, was also significant. Furthermore, the direct effect of depression on IGD symptoms (*b*_2_ = 0.76, SE = 0.28, *t* = 2.65, *p* < 0.05) was also statistically significant, whereas that of interpersonal relationships (path *b*_1_ in Figure 1) was not. Finally, no significant direct effect of impulsivity on IGD was found (*c*’ = 0.29, SE = 0.20, *t* = 1.42, *p* = 0.16) when impulsivity and both mediating variables were simultaneously entered into the equation. These results showed that interpersonal relationships and depression fully mediated the relationship between impulsivity and IGD symptoms in IGD group. Furthermore, the findings suggest that, in the IGD group only, high impulsivity leads to poor interpersonal relationships, and increasing depression, which in turn increases IGD symptoms. Additionally, the results of the bootstrapping to verify the indirect effects were significant only for the impact of impulsivity on IGD symptoms through interpersonal relationships and depression (B = 0.10, BCa 95% CI [0.0282, 0.2573]). In contrast, in the HC group, relationships between impulsivity, interpersonal relationships, depression, and IGD symptoms were not significant. A summary of the serial mediation results in the IGD group is shown in Table 3.

## 4. Discussion

In this study, we investigated the well-known association between impulsivity and IGD symptoms by examining a mechanism linking interpersonal relationships and depression by distinguishing between the IGD and HC groups. As no previous studies have examined the pathways between impulsivity, interpersonal relationship, depression, and IGD symptoms by distinguishing between IGD and HC groups, the main purpose of this study was to examine the mechanism of association among these variables. The findings revealed serial mediation effects of depression and difficulty with interpersonal relationships on the relationship between impulsivity and IGD symptoms in the IGD group, but not in HC group. Furthermore, there were only full mediation effects. These results suggest a pathway in the IGD group whereby high impulsivity was related to difficulty with interpersonal relationships, which increased depression and, thereby, the risk of IGD symptoms. Thus, impulsivity was identified as one of the reasons for problems with interpersonal relationships among IGD patients, and the effect on IGD symptoms is mediated by difficulties with interpersonal relationships and depression, rather than having direct effects on IGD symptoms. This result suggests that IGD symptoms can be alleviated by reducing interpersonal problems and depression. This conclusion should be considered in developing treatment programs for IGD patients who report high impulsivity. In particular, Cognitive-Behavioral Therapy (CBT), Interpersonal Psychotherapy of Depression, Acceptance and commitment (ACT), and group therapy might be included as an element of treatment programs to alleviate interpersonal problems and depression.

In addition, one of the main results of our study was those that differentiated between the IGD and HC groups. Participants with IGD were significantly more likely to be male. They reported significantly higher rates of IGD symptoms including internet gaming use time on weekdays and weekends, poor interpersonal relationships, high impulsivity, and depressive symptoms compared with the healthy control group. Bakken et al. [50] and Tsai et al. [51] found that male gender was a predictor of IGD symptoms, with a higher proportion of males in the IGD than in the normal group.

Choi (2012) [7] argued that IGD could be caused by bio-psycho-social factors. In keeping with that study, IGD symptoms were related to impulsivity as a biological factor, interpersonal relationships as a social factor, and depression as a psychological factor in the present study. Specifically, consistent with previous studies, our results showed significant relationships among IGD symptoms measured by the Y-IAT, depression, and difficulty in interpersonal relationships in both the IGD and HC group. Dalbudak et al. [14] found that depression, as measured by the Symptom Checklist-revised (SCL-90-R) was related to the risk for IGD. Similarly, IGD has been found to be associated with many psychiatric disorders and symptoms, including depression, anxiety, ADHD, hostility, interpersonal sensitivity, and paranoid ideation [32,33,41,52,53]. In addition, interpersonal relationships were also associated with IGD symptoms in our study, suggesting that poor interpersonal relationships are associated with IGD. Several previous studies have also found that interpersonal problems were known to cause IGD symptoms [24,31], and Caplan [54] found that people who had psychological problems preferred online interaction and used the internet to cope with loneliness. 

However, the relationship between impulsivity, including all subscales, and IGD symptoms was significant in the IGD group, but not in the HC group. In many previous studies, impulsivity was a significant risk factor for IGD [13,14,16,55]. In particular, high impulsivity has been shown to increase the severity of IGD symptoms; this relationship was also found in a study of Korean adults by Lee et al. [15]. However, our results showed that there was no significant correlation between impulsivity and IGD symptoms in HC group. In the previous studies, impulsivity differed significantly by gender, being more common among males [56,57]. The ratio of males to females in the HC group was significantly lower than that in the IGD group in this study. Therefore, the non-significant correlation between impulsivity and IGD symptoms in the HC group may reflect differences between groups in the gender ratio. 

There are several limitations to this study. First, it is difficult to generalize the results to other populations because this study was conducted with a small sample of young adults. Thus, it is necessary to include larger and more diverse samples in future studies. Second, this was a cross-sectional study, and all variables were measured at one point in time. MacKinnon et al. [58] suggested that longitudinal research would provide richer information about mediation and would be useful to identify causal relationships when analyzing mediation effects. Therefore, future studies should be longitudinal study so that clear pathways and causal relationships could be more readily identified.

## 5. Conclusions

The findings of this study clarify the main factors to be considered when intervening in IGD, particularly in the case of IGD patients with high impulsivity. Additionally, the present findings highlight the importance of the role of bio-psycho-social factors in relation to IGD symptoms. Thus, we should focus on reducing interpersonal problems and depression when performing interventions to improve IGD symptoms in individuals with high impulsivity.

## Figures and Tables

**Table 1:** Demographics and Clinical characteristics (*n* = 118).

Variables	IGD (*n* = 62) M ± SD	HC (*n* = 56) M ± SD	$\chi^{2}/t$	*p* Value
Gender (male, %)	55 (88.7%)	38 (67.9%)	7.662 *	0.007
Age (years)	25.54 ± 5.29	24.23 ± 3.92	1.545	0.125
Education (years)	13.88 ± 1.70	13.92 ± 1.51	−0.139	0.890
Time for weekday (h/day)	3.89 ± 3.02	0.28 ± 0.73	9.088 **	<0.001
Time for weekend (h/day)	5.72 ± 3.53	0.44 ± 1.09	10.979 **	<0.001
Y-IAT	53.71 ± 15.93	30.34 ± 9.15	9.855 **	<0.001
RCS	83.43 ± 14.48	94.87 ± 9.99	−5.033 **	<0.001
BIS-11	64.87 ± 9.65	57.33 ± 7.59	4.673 **	<0.001
BIS-11_Cognitive	19.03 ± 2.98	17.60 ± 2.39	2.840 *	0.005
BIS-11_Motor	18.09 ± 4.12	14.17 ± 2.84	6.053 **	<0.001
BIS-11_Nonplanning	27.74 ± 4.75	25.55 ± 3.82	2.735 *	0.007
BDI	12.04 ± 8.58	4.28 ± 4.30	6.261 **	<0.001

* *p* < 0.05, ** *p* < 0.001. Time for weekday/weekend = Average internet gaming use time per day on weekday and weekend; IGD = Internet Gaming Disorders; HC = Healthy Control; Y-IAT = Young’s Internet Addiction Test; RCS = Relationship Change scale; BIS-11 = Barratt Impulsiveness Scale; BDI = Beck Depression Inventory.

**Table 2:** Correlation Analysis among the variables in IGD and HC group.

**IGD (*n* = 62)**	**Y-IAT**	**BIS-11**	**BIS-11_Cognitive**	**BIS-11_Motor**	**BIS-11_Nonplanning**	**BDI**	**RCS**
Y-IAT	1						
BIS-11	0.306 *	1					
BIS_cognitive	0.375 **	0.783 **	1				
BIS_Motor	0.129	0.765 **	0.388 **	1			
BIS_Nonplanning	0.275 *	0.875 **	0.626 **	0.441 **	1		
BDI	0.472 **	0.334 **	0.407 **	0.136	0.308 *	1	
RCS	−0.285 *	−0.407 **	−0.487 **	−0.103	−0.432 **	−0.641 **	1
**HC (*n* = 56)**	**Y-IAT**	**BIS-11**	**BIS-11_Cognitive**	**BIS-11_Motor**	**BIS-11_Nonplanning**	**BDI**	**RCS**
Y-IAT	1						
BIS-11	−0.023	1					
BIS-11_cognitive	0.070	0.744 **	1				
BIS-11_Motor	0.062	0.845 **	0.489 **	1			
BIS-11_Nonplanning	−0.136	0.893 **	0.489 **	0.630 **	1		
BDI	0.393 **	0.340 *	0.414 **	0.369 **	0.143	1	
RCS	−0.323 *	−0.317 *	−0.453 **	−0.289 *	−0.133	−0.586 **	1

* *p* < 0.05, ** *p* < 0.001. IGD = Internet Gaming Disorders; HC = Healthy Control; Y-IAT = Young’s Internet Addiction Test; RCS = Relationship Change scale; BIS-11 = Barratt Impulsiveness Scale; BDI = Beck Depression Inventory.

**Table 3:** Summary of serial mediation analysis of interpersonal relationship and depression between impulsivity and IGD symptoms in IGD group. (*n* = 61, bootstrap = 5000).

Effect	Paths	B	SE	*t*	BCa 95% CI	
					Lower	Upper
Direct effects	Impulsivity → Relationship	−0.60	0.17	−3.39 *		
	Impulsivity → Depression	0.06	0.09	0.72		
	Relationship → Depression	−0.35	0.06	−5.53 **		
	Relationship → Internet Addiction	0.05	0.16	0.30		
	Depression → Internet Addiction	0.76	0.28	2.65 *		
	Impulsivity → Internet Addiction	0.29	0.20	1.42		
Indirect effect	Impulsivity → Relationship → Internet Addiction	−0.01	0.07		−0.1759	0.1243
	Impulsivity → Relationship → Depression → Internet Addiction	0.10	0.05		0.0282	0.2573
	Impulsivity → Depression → Internet Addiction	0.03	0.04		−0.0268	0.1514

* *p* < 0.05, ** *p* < 0.001. BCa = Biased-Corrected and Accelerated 5000 bootstrapping; Covariate = sex.
